# Community perceptions and practices of treatment seeking for childhood pneumonia: a mixed methods study in a rural district, Ghana

**DOI:** 10.1186/s12889-016-3513-z

**Published:** 2016-08-22

**Authors:** Mercy Abbey, Margaret A. Chinbuah, Margaret Gyapong, L. Kay Bartholomew, Bart van den Borne

**Affiliations:** 1 Research and Development Division, Ghana Health Service, PM Bag 190, Accra, Ghana; 2 Dodowa Health Research Centre, Ghana Health Service, P.O. Box 1, Dangme-West District, Ghana; 3 School of Public Health, University of Texas Health Science Centre, 1200 Herman Pressler, Suite W238, Houston, TX 77030 USA; 4 Department of Health Promotion, University of Maastricht, P.O. Box 616, Maastricht, 6200 MD The Netherlands

**Keywords:** Caregivers, Children under five, Care seeking, Home management, Perceptions, Pneumonia, Ghana

## Abstract

**Background:**

The World Health Organization recommends community case management of malaria and pneumonia for reduction of under-five mortality in developing countries. Caregivers’ perception and understanding of the illness influences the care a sick child receives. Studies in Ghana and elsewhere have routinely shown adequate recognition of malaria by caregivers. Similarly, evidence from Asia and some African countries have shown adequate knowledge on pneumonia. However, in Ghana, little has been documented about community awareness, knowledge, perceptions and management of childhood pneumonia particularly in the Dangme West district. Therefore this formative study was conducted to determine community perceptions of pneumonia for the purpose of informing the design and implementation of context specific health communication strategies to promote early and appropriate care seeking behaviour for childhood pneumonia.

**Methods:**

A mixed method approach was adopted. Data were obtained from structured interviews (*N* = 501) and eight focus group discussions made up of 56 caregivers of under-fives and eight community Key Informants. Descriptive and inference statistics were used for the quantitative data and grounded theory to guide the analysis of the qualitative data.

**Results:**

Two-thirds of the respondents had never heard the name pneumonia. Most respondents did not know about the signs and symptoms of pneumonia. For the few who have heard about pneumonia, causes were largely attributed to coming into contact with cold temperature in various forms. Management practices mostly were self-treatment with home remedies and allopathic care.

**Conclusion:**

The low awareness and inadequate recognition of pneumonia implies that affected children may not receive prompt and appropriate treatment as their caregivers may misdiagnose the illness. Adequate measures need to be taken to create the needed awareness to improve care seeking behaviour.

**Electronic supplementary material:**

The online version of this article (doi:10.1186/s12889-016-3513-z) contains supplementary material, which is available to authorized users.

## Background

Malaria and pneumonia are leading causes of childhood morbidity and mortality in Sub Saharan Africa (SSA) killing approximately 1.4 million children every year [1]. Although effective drugs are available, many of the affected children do not receive treatment within the first 24 h period after onset of symptoms [2, 3]; and many of them die at home [4].

To increase access to prompt and appropriate treatment for ill children, community-based interventions, mostly involving Community Health Workers (CHWs) have been introduced in areas that lacked access to formal health facilities [5]. CHWs are laypersons selected by their communities to render some basic health services within the communities, after undergoing short-term training [6]. Early examples of such community-based service delivery strategies include the home management of malaria (HMM) which provided anti-malaria medication for the treatment of fever presumed to be malaria in children under five [7]. Though HMM led to prompt treatment of fever due to malaria, other febrile non-malaria illnesses or conditions (including pneumonia) were often inappropriately treated with anti-malarial only, resulting in delayed treatment for pneumonia [8].

In 2004, The World Health Organization (WHO) and the United Nations Children Fund (UNICEF) supported recommendations for community level treatment of pneumonia as part of integrated community case management (ICCM) activities in areas where malaria and pneumonia are endemic [3]. Several countries, including Ghana have subsequently adopted this strategy. CHWs in Ghana have been used successfully in community health service delivery programs (mostly pilot studies) targeting single diseases such as malaria. Previous attempts to train and deploy CHWs in management of childhood pneumonia were unsuccessful and therefore abandoned due to poor performance in differentiating malaria from pneumonia for appropriate treatment [9]. Treatment of childhood pneumonia has therefore been restricted mainly to health facilities. Following the recommendation on community case management of pneumonia, a pilot randomized controlled trial was implemented in the Dangme West district in Ghana to operationalize the strategy and to explore the effect of community management of pneumonia and malaria on under-five mortality in the study district.

The effectiveness of such community level interventions requires the utilization of the services by caregivers of the sick child. The utilization of such services by the caregivers may be influenced by the caregiver’s perception and understanding of the illness [10], it is therefore pertinent to assess the caregivers’ perceptions of childhood pneumonia and their treatment seeking practices for the sick child. Community perceptions of malaria and treatment seeking behaviour have been widely studied. For example, Studies in Ghana and elsewhere have shown that mothers had knowledge to recognize symptoms suggestive of malaria [11–14]. Similarly, studies conducted on pneumonia in countries including Pakistan, Peru and Kenya showed that most caregivers were aware of and could easily recognize pneumonia signs and symptoms [15–17]. However, in Ghana, little has been documented about community awareness, knowledge, perceptions and management of childhood pneumonia particularly in the Dangme West district. Therefore, this study was conducted to determine community perceptions of pneumonia for the purpose of informing the design and implementation of context specific health communication strategies for appropriate care seeking behaviour for childhood pneumonia. In addition, it examines what factors are related to caregivers’ willingness or intention to use CHW services for fever in children under five in the Dangme West district.

## Methods

### Study site

This study was carried out in the Dangme west district of Greater Accra region in Ghana. At the time of the study, the district had an estimated land size of 1,700 square kilometres with a population of about 109 459 and 376 communities or villages [18]. Available health facilities at the time of the study included four government owned health centres, six community clinics, two privately owned clinics, two private maternity homes, two pharmacies, and 42 registered drug retail shops. The district had no hospitals. Severe cases were referred to hospitals in neighbouring districts. The indigenes are of the Ga-Adangme ethnic group and the dominant occupation is trading followed by subsistence farming.

### Study design

The study employed a mixed methods design to explore community perceptions and practices in the management of childhood fevers with emphasis on respiratory symptoms (especially pneumonia).

### Study population and sample

#### Key informant interviews and focus group discussions

We conducted a total of eight focus group discussions (FGDs), with seven female groups and one male group, made up of 56 parents and caregivers of under-fives. A caregiver was defined as persons having the primary responsibility of caring for an under-five child. This person could be a male or female biological parent or guardian of the child. In this paper, the words parent(s)/caregiver(s) are used interchangeably. We carried out in-depth, key Informant Interviews (KIIs) with three Traditional Birth Attendants (TBAs), one herbalist, three chemical shop attendants and one community leader as key Informants. We defined a Key Informant as a community member seen by peers as an authority, an opinion leader or a well-known community member having adequate knowledge to discuss issues related to the inhabitants; including health issues and health seeking behaviour in the community. The numbers of FGDs and KIIs were deemed sufficient when subsequent discussions and interviews yielded little or no new information [19].

### Household survey

In addition to the KI interviews, we conducted a household survey of a representative sample of 501 caregivers. Details of the sample were accessed via the database of the Dodowa Health and Demographic Surveillance System (DHDSS). This database comprises of information on socio-demographic characteristics of the district population; such as age, sex, marital status and total number of children per woman. Other variables are a unique identification code for each caregiver, community of residence and house address.

The respondents were selected through a multistage sampling technique as follows: 15 communities were randomly selected from each of the four sub districts. All houses within the selected communities with under-fives were selected, and from each selected house, only one eligible caregiver from a household was selected for interview, by simple random sampling.

### Procedures: interviews and focus groups

We purposively recruited key Informants and FGD participants with the assistance of a community mobilization officer who lived in the community. Research team members screened recruits for eligibility and selection prior to the day of discussion. The discussions were held in quiet places within the communities and in the local language, Dangme, to allow participants to express themselves more easily and to elicit local terminology that could be useful in designing messages for the intervention. A social scientist who spoke Dangme fluently conducted the Key Informant interviews and moderated the focus groups. She was assisted by a note taker who was proficient in Dangme and the first author who only had basic knowledge of the language. A list of topics, complemented with a video on Acute Respiratory illnesses (ARI) for training health workers in Integrated Management of Childhood Illnesses (IMCI) was used to guide the discussions. Topics discussed included caregiver perceptions of common childhood illnesses in their communities, breathing difficulties among children, recognition of childhood pneumonia, the local name for pneumonia and its treatment seeking practices (Assessment of pneumonia was based on IMCI criteria i.e. the presence of cough, difficult or fast breathing with or without fever). The video sections showed a child suffering from pneumonia; with fast breathing and chest in drawing. The use of the video was to help participants see the illness symptoms and to generate more terms related to the illness from the participants [20]. The discussions were audio recorded with consent from participants. Each discussion lasted about one hour to one hour thirty minutes.

The qualitative data was reviewed to extract repeated ideas or themes that were apparent. Themes that emerged from the key Informants and focus group discussions were not only grouped into categories but were further used to design the survey instrument.

The household survey assessed caregiver perceptions, and practices in the management of fevers and breathing difficulties with emphasis on pneumonia in children under five. Information sought from the respondents included caregivers’ perceptions of common childhood illnesses in the communities, perceptions of severe childhood illnesses, recognition of the symptoms and causes of pneumonia, treatment seeking practices for the disease and the local name for pneumonia. Other variables covered demographic characteristics of the caregivers and whether or not caregivers were willing to utilize CHW services for treatment of fever in under-fives if made available and actions taken at home for childhood fevers.

A team of eight trained research assistants (Six interviewers and two serving as supervisors) conducted face to face interviews with caregivers in their homes. Interviews were held in the local languages using a questionnaire with both open and closed ended questions. Several role play sessions of translating questions from English to the local languages and field pretesting among the data collection team ensured adequate practice prior to actual field data collection. Supervisors helped to resolve any emerging challenges during the field data collection and also checked filled questionnaires for completeness. Data collection lasted four weeks for both qualitative and quantitative survey.

### Data analysis

The household data was coded, categorized, entered in SPSS and was exported and analyzed in STATA. Descriptive and inference statistics were the primary tools used to explore and interpret findings of the quantitative survey data. To examine the factors that determine the use or otherwise of CHW services, probability (probit) estimation technique was employed for this study because it uses maximum likelihood estimation. Furthermore, the dependent variable is a binary dummy variable which takes value of 1 if CHW services are used and 0 if CHW services are not used [21]. The dependent variable in this study is the intention to use the services of the CHWs or not. The explanatory variables included the age of the caregiver, sex, religion, education, constraints (financial, unfriendly staff, and proximity to the CHW), marital status and birth parity (number of children) of the respondents.

The tape-recorded FGDs and KIIs were transcribed verbatim, supplemented with field notes and any identifying information removed to ensure participants anonymity [19]. We adopted the grounded theory approach to the data analysis. The first author and the moderator of the interviews read all transcripts several times to familiarize with the data, identify key themes and develop a coding scheme. The transcripts were analyzed and coded. We compared coded themes and discussed discrepancies until agreement was reached. We then grouped segments of the interviews under the relevant codes and further analyzed the data by sub themes.

## Results

Results are presented by methods. We report first on results of the quantitative study then the qualitative. Key findings reported are demographic characteristics, perceptions on pneumonia and treatment practices, intention or willingness to use CHW services and treatment for childhood fever.

### Demographic characteristics of respondents

The caregivers were predominantly female, and aged between 25 and 34 years, as shown in Table 1. Twenty nine percent of the respondents had no formal education. Most were Christians (87.6 %) and married (87.8 %).

**Table 1:** Demographic Characteristics of Caregivers in Selected Households in Dangme West District, Ghana

Variable	*N* (501)	Percent
Sex		
Male	53	10.6
Female	448	89.4
Age (Years)		
16–24	105	20.9
25–34	234	46.7
35–44	103	20.6
45–54	35	7.0
55+	24	4.8
Religion		
None	23	4.6
Christianity	439	87.6
Muslim	32	6.4
Traditionalist	5	1.0
Missing	2	0.4
Education		
None	144	28.7
Primary	138	27.5
JSS/Middle School	184	36.7
Secondary/SSS	21	4.2
Tertiary	11	2.2
Vocational/Technical	3	0.6
Ethnicity		
Ga Adangme	341	68.1
Ewe	123	24.6
Akan	15	3.0
Dagomba/Gonja/Mamprusi	15	3.0
Others	5	1.0
Missing	2	0.4
Marital status		
Never Married	11	2.2
Married	440	87.8
Divorced/separated/widowed	50	10.0
Occupation		
Unemployed	83	16.6
Farmer	124	24.8
Artisan	81	16.2
Trader	181	36.1
Others	31	6.2
Missing	1	0.2

### Perceptions on pneumonia

The caregivers’ knowledge about pneumonia is shown in Table 2. The results suggest that, perceptions and understanding of illnesses that affect under-fives, and adequate knowledge about their causes, effects and treatments are context specific. Most respondents were inadequately informed about pneumonia, its signs, symptoms, causes and treatment. Only a third of respondents 31.94 % (160/501) indicated they had ever heard the name pneumonia. Of those who reported having ever heard of pneumonia, majority 58.13 % (93/160) did not know any symptoms of pneumonia as shown in Table 2. Only 5.62 % (9/160) mentioned a word in Dangme for pneumonia and each name varies.

**Table 2:** Reported knowledge on pneumonia among caregivers in Dangme West district, Ghana

Variable	(Yes) *n*	Percent	(No) *n*	Percent	Don’t know (*n*)	Percent
Heard of pneumonia (*N* = 501)	160	31.94	341	68.06	-	
Signs and symptoms of pneumonia (*N* = 160)						
Fever	18	11.25	49	30.63	93	58.12
Fast breathing	18	11.25	49	30.63	93	58.12
Difficulty breathing	19	11.88	48	30.00	93	58.12
Cough	23	14.38	44	27.50	93	58.12
Causes (*N* = 160)						
Sleeping on bare cemented or cold floor	59	36.87	26	16.25	75	46.88
Exposure to cold weather	56	35.00	29	18.12	75	46.88
Sleeping close to electronic r fan	28	17.50	57	36.61	75	46.88
Eating cold food/drinking very cold water	13	8.12	72	45.00	75	46.88
Treatment for pneumonia (*N* = 160)						
Take the sick child to the health facility	92	57.50	-	-	68	42.50
Can pneumonia be prevented? (*N* = 160)						
Yes	97	60.63	2	1.25	61	38.12
Preventive measures/ways to prevent pneumonia (*N* = 160)						
Avoid sleeping on the bare cemented floor	59	36.88	25	15.62	76	47.50
Avoid exposure to cold weather	54	33.75	30	18.75	76	47.50
Avoid eating cold food/very cold water	14	8.75	70	43.75	76	47.50
Avoid sleeping with fan on	28	17.5	56	35.00	76	47.50

### Intention or willingness to use CHW services

Majority, 96.6 % affirmed their willingness to utilize the CHW services for their children if such services are made available in their communities. The probit regression showed that age, education, religion, sex and occupation are not important predictors of the intention (willingness) of caregivers to use services of the CHWs for childhood fever. Marital status and birth parity however significantly influence a caregiver’s decision to use services of the CHWs (Table 3). Married respondents were more likely than the unmarried to use services of the CHWs (*P* < 0.001). The use of these services also increases with an increasing parity (*P* < 0.001). Financial challenges and proximity to the CHW’s location are some reasons why respondents may not use the services of CHWs.

**Table 3:** Probit regression results for intention to use services of CHWs and background characteristics

Intention to use the services of CHW	Coefficient	Standard Error	z	*P* > z
Age of respondent	0.016	0.015	1.06	0.29
Female (Reference: Male)	0.582	0.302	1.75	0.081
Married (reference: not married)	0.917	0.25	3.67	0.000^a^
Birth Parity (Number of Children)	1.416	0.233	6.09	0.000^a^
Education (Reference: No education)				
Primary	0.055	0.385	0.14	0.887
JHS	−0.448	0.348	−1.29	0.199
Secondary plus	−0.593	0.532	−1.11	0.265
Reason for not using (Reference: financial)				
Unfriendly staff	0.124	0.374	0.33	0.74
Distance/proximity	0.808	0.326	2.48	0.013^b^
Religion (Reference: None)				
Christian	−0.683	0.68	−1	0.315
Moslem	−0.539	1.434	−0.38	0.707
Occupation (Reference: Unemployed)				
Farmers	−0.0689	0.386	−0.18	0.859
Artisans	0.0412	0.390	0.11	0.916
Traders	0.4860	0.352	1.38	0.167
Formal sector of the economy	−0.2545	0.693	−0.37	0.713
Employed (other jobs)	−0.2769	0.591	−0.47	0.639
_cons	−0.6888	0.886	−0.78	0.437

^a^Significant at 1 %, ^b^significant at 5 %

### Treatment seeking practices for fever

The respondents gave a clear description of various treatment practices for fever. As shown in Table 4, the child is usually given a tepid sponging (61.9 %) to bring down the temperature, followed by self-administered home treatment with remnants of orthodox medicine kept from a previous ailment or herbal remedies. The common medication administered at home were mostly analgesics such as paracetamol (92.4 %) and anti-malarial, mainly chloroquine (33.1 %). Majority, (93.4 %) would normally initiate treatment on the same day or within 24 h from when symptoms are observed.

**Table 4:** Caregiver management of fevers in under 5 s in Dangme west district (*N* = 501)

Variable	Yes n (%)	No n (%)
Action taken at home/Treatment given for child’s fever		
Tepid sponging of the child	310 (61.9)	191 (38.1)
Use of left over medicine available at home	293 (58.5)	208 (41.5)
Seek care from over –the –counter medicine seller	103 (20.6)	398 (79.4)
Seek care from a health facility	65 (13.0)	436 (87.0)
Give herbal medicine	21 (4.2)	480 (95.8)
Give enema at home	11 (2.2)	490 (97.8)
Seek care from a CHW	0 (0.0)	501 (100.0)

### Background characteristics of FGD participants

Participants in the FGDs comprised of young mothers, older mothers, grandmothers and fathers. The young mothers were aged between 21 and 32 years and had one to three children, some were married. Many of these mothers have primary and/or Junior Secondary School education while few of them have no formal education. They had lived in the community most of their lives. Most of these younger mothers were traders or artisans. A few were unemployed.

Older mothers were aged between 35 and 47 years. Most were married while some were divorced or single parents. Almost all the older mothers had little or no formal education and had lived in the community since birth. The grandmothers were engaged in either farming or trading or both, were averaged between 50 and 77 years, and had lived in the community most of their life time.

Men who were engaged in the FGDs were between the ages of 20 and 43 years and were mostly married. All male participants were employed, and had lived in the community between 10 and 38 years. Most of them were farmers and the rest artisans. All of them had some formal education with the highest being secondary education.

Key issues explored from the various study participants in the qualitative interviews yielded similar responses therefore the findings are presented together and under three main themes: Community perception of common childhood illnesses; perceived causes of difficulty in breathing and or pneumonia and Treatment seeking for these illnesses.

### Perceptions of common childhood illnesses among under-fives

Key Informants and caregivers perceived malaria, ‘hiowe’ or ‘ablam’ (convulsion), ‘*haem-lar-dum*’ or ‘hedola’ (hot body/fever) diarrhea and measles as the common causes of under-five mortality in the district. Other conditions perceived as non-life threatening were, stomach pains, skin rashes, cough and worm infestation.

On the whole, “difficulty in breathing or breathing difficulties” was rarely mentioned spontaneously as one of the common illnesses among children in the district. Further probing revealed that difficulty in breathing in children was often associated with “soso” (common cold) or “blocked nose”, “hoha” (asthma) and convulsion.

However, after FGD participants and KIs viewed the video of a child with high respiratory rate, cough and lower chest in-drawing, indicating severe pneumonia (WHO’s IMCI classification), majority of the discussants generally described the child in the video as having breathing problems. Very few (*about 11 % of 56 of FGD participants*) including a herbalist spontaneously identified the child as suffering from pneumonia. A male participant in one of the FGDs exclaimed “…*oh that is pneumonia; it is common among adults but not the children*”.

In another FGD session, a female participant also explained, *“…That is pneumonia; because pneumonia causes a child to cough and breathe like this.”*

When probed further, majority said they have never heard the word “pneumonia” before, adding that it was their first time of hearing it. Others, who reported they have heard about pneumonia, indicated the radio, school and clinic as their source of information.

It appeared that there was no single word or expression used to describe “pneumonia” in the local dialect. Various expressions used in the Dangme dialect to describe the condition included “*emumu-sue-no*” or “*emumu-siueo-nowie*” (meaning “gasping for breath”) and “*emumu-dei-no*” or “*enge-mumu woe mla mla mla*” (meaning “the child is breathing faster than normal”). The expressions given by respondents in the household survey included: “… *kasa hio”* (meaning “illness of the rib area”); *“fie se ehem*” (meaning “cold air has entered into his/her body”).

Additionally, the herbalist, who identified the child as suffering from pneumonia, suggested the name for pneumonia in the local dialect as *“Mumunya tam hio”* (meaning, “out of breath” illness). The variety in common expressions among the participants suggest inadequate exposure/knowledge or understanding of pneumonia except for those who have ever seen someone suffer from it or someone who had been diagnosed of it as told by one female participant. She said *“I went to visit my relative on admission to the hospital and she told me the Nurse told her that she had pneumonia…she was not breathing properly but I don’t know a name for it in Dangme”*.

### Perceived causes of breathing difficulties and or pneumonia

Most participants perceived causes of breathing difficulties in children as common cold, headache, cough, asthma, hot body and ‘hidden measles’ (this is when a child has other symptoms of measles without the skin rash), eating ‘Bad food,’ (junk food) and being infested with worms and measles.

The perception of caregivers who have heard about pneumonia differed. The general belief was that exposing a child, to cold temperature or windy weather conditions could result in the child getting pneumonia. The most common factors mentioned in all discussion groups included “*sleeping on the bare cemented floor*”. For instance, one female participant said *“What I know is that pneumonia is caused by sleeping on the bare cemented floor; because the bare floor is cold, if the child is not adequately clothed and sleeps on it for a long time, the cold penetrates the body and causes pneumonia.”*

Another belief expressed by a male discussant was that, *“Sleeping directly under a fan can cause a child to have pneumonia*; *I mean the continuous exposure of a child to the air from an electric fan over a long period of time makes the child sick with pneumonia*.”

Furthermore, other views sampled attributed the cause of pneumonia to ingesting cold drinks or food; they indicated that, *“One gets pneumonia from drinking cold water,* eating *cold food or chewing ice cubes*.” The Herbalist also added that, “… *In the past, when there was no iced water children did not get pneumonia.”*

Poor hygiene practices was also mentioned as one of the factors that could cause the child to get pneumonia; as explained by a male participant, *“When a child picks anything from the ground and puts it in his mouth, there can be contamination which can cause the child to have pneumonia. Crawling children get it easier than toddlers.”*

### Treatment seeking practices for difficulty in breathing and pneumonia

Overall, treatment practices that were mentioned by participants can be grouped into Self-treatment and Hospital treatment.

#### Self-treatment

Self-treatment refers to a situation where a person determines what medicine, as well as the dosage to use for an ailment without consultation with a qualified health personnel. The Self-treatment practiced by community members were in the form of home remedies including the use of Shea butter, honey and herbal treatments. Discussants believe Shea butter has medicinal properties and can be used both externally and internally. Some uses mentioned include massaging the sick child’s rib area with the Shea butter, particularly by a woman who has ever given birth to twins. The belief is that such persons possess special supernatural abilities that make the massage more effective and facilitates the healing process. Another approach is to make a mixture of Shea butter, herbs and/or honey for the massage. For internal usage, ingesting or inhaling melted Shea butter is believed to clear the nasal congestion and restore proper breathing as explained by one female participant in FGD; *“Catarrh also causes the child to have difficulty in breathing so when my child gets “catarrh” I normally put shea butter into the nose and give him some to eat then the blockage opens and the child begins to breathe properly.”*

Another common form of treatment mentioned was massaging the child with Robb methylated ointment and hot water. This is particularly done for children with pain in the rib cage area. A less common practice indicated by few caregivers is to give the child water drained from soaked uncooked rice, or honey to treat the difficulty in breathing as cited by one grandmother in a FGD; *“We soak rice in water and give the cloudy water to the child to drink. This cleans up the child’s system and health is restored.”*

Herbs in different forms were reportedly used for treatment of pneumonia in children. Some are boiled or made into powder and mixed with certain substances obtained from a herbalist for use as described by one male participant “*The herbs are boiled and given to the child to drink or given as enema. This flushes out the illness through the passing of stool.”*

The herbalist also revealed that, *“There is one called “Ti” which is also smeared on the child’s body. “Ti” is black powdered substance processed from a combination of herbs and other ingredients made into powder and this is given to the child who has fast breathing problems for remedy.”*

#### Hospital treatment

The other major form of treatment for difficulty in breathing was sought at the hospital as some considered it difficult for home treatment. This approach was common among caregivers within and closer to the district capital where access to facility treatment was easy and convenient. Some Caregivers expressed that they would usually initiate some form of treatment at home but most invariably, the sick children ended up in the clinic. One of such views pertained to treatment for asthma as mentioned by one female participant; *“For asthma, first, we give honey and M & B (an anti-biotic) at home. If the child does not respond well to the medicine, then we send the child to the clinic for further treatment.”*

Others seek medication for breathing difficulties directly from drug shops in the community as narrated by one drug shop attendant:

*“… people do come here (drug shop) complaining about breathing problems. They would say for example, the way my child is breathing, the child is having asthma. Normally when they behave like that we advise them to take the child to the hospital first because there are different drugs for asthma so when they visit the hospital and the drug is prescribed for them they come back to buy.”*

### Decision making on treatment seeking for the sick child

Discussants indicated that, decisions on treatment seeking are most often taken in a consultative manner. The immediate caregiver consults relatives or neighbours for ideas on the best course of treatment for the illness. It often involved seeking the opinion or assistance of significant others who are considered knowledgeable in managing the child’s illness. Such persons included grandparents of the child, other older relatives and neighbours as explained by a female participant: *“I don’t know any medicines for any illness and since my parents are older than I am and they know what is wrong with the child they take the decision on what medicine to give to the child.”*

Nonetheless, there are instances where the decision rests on the caregiver present at the time when the illness is observed as pointed out by one male participant: *“My wife is the one who takes care of the children when I am not in the house, but when I am in the house I take the decision on what to do.”*

## Discussion

This study aimed to assess caregivers’ awareness and perceptions of pneumonia, its causes, signs and symptoms and their treatment seeking practices for children with the symptoms of pneumonia. The mixed method approach proved useful in confirming perceptions expressed in the quantitative and qualitative responses. In-depth interviews and focus group discussions provided explanation of the quantitative responses, which validated knowledge gathered from the responses as true reflections of opinions and beliefs of the respondents.

The study showed that caregivers were aware of various common childhood illnesses prevailing in their communities; however, pneumonia did not emerge as one of the known common childhood diseases in their communities. The biomedical symptoms indicative of pneumonia including fast breathing, difficult breathing and cough were not mentioned spontaneously or associated with pneumonia even when prompted.

The seeming low awareness of pneumonia among the rural dwellers of Dangme West district is consistent with earlier findings of studies conducted in Eastern Uganda among mothers and traditional healers [22] but in contrast to the findings in Kenya [16], Pakistan [15], Peru [17] and Western Uganda [23], where participants had heard about the illness and were easily able to identify the signs and symptoms. It can therefore be posited that such problems of low awareness are community specific, and may be dependent on the linguistic interpretation of diseases and how they may be perceived within the communities. This became evident from the video illustration of the sick child, where participants identified and associated pneumonia with varied local terminologies but could not agree on a known local name; as such, there are some caregivers that have heard the term “pneumonia” but do not understand what it is. Similarly, findings from Eastern Uganda showed that participants had local names for the symptoms but no specific name for pneumonia [22] which is in contrast to study findings in Kenya [16] and Pakistan [15] that reported knowledge of specific local names for pneumonia and its symptoms among study participants. There may also be a possibility that the people of Dangme West district, a primarily rural area have had very little education or information on pneumonia and its symptoms.

This lack of ability among caregivers in the present study to identify pneumonia symptoms could result in possible delays in seeking appropriate health care for the sick child and result in avoidable deaths [24].

Treatment practices for breathing difficulties and or pneumonia, cited by caregivers in the present study and also reported in other studies in Pakistan [15] and Uganda, [22] concur and resonates with common practises for managing childhood illnesses. Many caregivers in Sub Saharan Africa try different home remedies and resort to facility treatment after self-treatment efforts have failed and or illness worsens [11, 25]. On the other hand, a study in Kenya found no known home treatment for pneumonia but rather emphasized hospital treatment [16].

We note an interesting observation in the relationship between causes, prevention and treatment of pneumonia in the current study and that of Peruvian mothers: Caregivers in Dangme west district employed mostly herbal, hospital treatment or some home remedy to treat Pneumonia, mothers studied in Peru had one peculiar form of treating pneumonia. As they had more knowledge on the causes of pneumonia, they considered doing the opposite of the causative factor, as a form of treatment [17]. For instance, *“*If cold temperature was indicated as one important cause of pneumonia, then making the child sweat or providing heat will bring health.” As a result, Peruvians explored practices like sauna, spa and thermo therapeutic methods as a ways of treating some ARIs.

In the Ghanaian study, a similar approach can be seen; though the respondents in Ghana, unlike their Peruvian counterparts, rather perceived the opposite of the causes of pneumonia as a way of preventing the illness altogether. For example, if sleeping on the bare cemented floor caused pneumonia, then they mentioned ‘avoiding sleeping on bare cemented floors’ as a preventive measure. This is evident in Table 2 where the various causes of pneumonia mentioned was the same as the various preventive measures, which were actually the reverse of its causes. From the responses, it could be deduced that if caregivers in the present study were convinced that bacteria or virus causes pneumonia, then they would seek appropriate protection and care.

This study found proximity to a CHWs’ location as a significant factor for the willingness to utilize the CHW for the child’s fever (*p*-value = 0.013). This means that CHW services have a high probability of being used if they are located closer to the communities they serve and this can be explained by the fact that proximity to the service provider has advantages including reduction in cost for transportation, waiting time and elimination of other costs associated with health seeking at health facilities. This result is similar to findings reported by other researchers [18, 26, 27].

### Limitations

Assessing actual behaviour of study participants would have been more ideal than exploring perceptions of participants since this may have revealed their actual practice. The use of a triangulation of quantitative and qualitative methods with different participants however strengthened our findings from the different community perspectives explored. The use of the video showing real children with pneumonia proved useful. It enriched the discussions as it elicited more information from participants compared with the use of the discussion guide alone [20]. The study findings could be largely generalized to the district due to the probability sampling method used in selecting respondents for the household survey.

## Conclusion

Majority of caregivers in this study were unable to recognize or identify symptoms indicative of pneumonia. Most could not appreciate its dangers or identify it as a deadly childhood disease and as a result take appropriate steps to manage a child affected by it. Intense education at the home and community level, through appropriate behaviour change communication methods and targeted at various stakeholders is necessary to increase awareness and to improve care seeking behaviour. As a result of this formative study, particularly due to the low awareness of pneumonia, and given that caregivers could not distinguish between pneumonia and malaria, we changed our original intent of specifying malaria and pneumonia in our campaign messages. We refined the message to reflect that fever can be a sign of several illnesses and that a child observed to have fever, should be taken immediately to the CHW or the nearest health facility for appropriate care.

This strategy was more amenable to promoting prompt and appropriate care seeking rather than requiring caregivers to attempt a diagnosis at home before seeking appropriate care for the sick child.

## Additional file

Additional file 1: Community perceptions of pneumonia Data set. (XLS 311 kb)
